# Endocannabinoids and Reproduction

**DOI:** 10.1155/2014/378069

**Published:** 2014-01-20

**Authors:** Rosaria Meccariello, Natalia Battista, Heather B. Bradshaw, Haibin Wang

**Affiliations:** ^1^Dipartimento di Scienze Motorie e del Benessere, Università di Napoli Parthenope, 80133 Naples, Italy; ^2^Faculty of Bioscience and Technology for Food, Agriculture and Environment, University of Teramo, 64100 Teramo, Italy; ^3^European Center for Brain Research (CERC)/Santa Lucia Foundation, 00143 Rome, Italy; ^4^Department of Psychological and Brain Sciences, The Kinsey Institute for Research in Sex, Gender and Reproduction, Indiana University, Bloomington, IN 47405, USA; ^5^State Key Laboratory of Reproductive Biology, Institute of Zoology, Chinese Academy of Sciences, Beijing 100101, China

Infertility is a worldwide reproductive health problem whose consequences have deep psychological and social impact in health, demographic change, and wellbeing. Thus, the knowledge of basic, conserved modulators of reproduction might contribute to the discovery of new potential target for the exploitation of drugs to treat infertility in humans. Besides the well known effects of endocannabinoids in the control of pain and visceral functions, in the last decade the deep involvement of endocannabinoid system in the control of reproductive functions in both males and females emerged. In fact, endocannabinoids, endogenous lipids that bind to cannabinoid receptors, modulate reproductive axis at both central and local level. Endocannabinoid signalling is critical for gonadotropin release and sex steroid biosynthesis, for the formation of functional male and female gametes, for fertilization, preimplantation embryo development, implantation, and postimplantation embryonic growth, and for labouring delivery as well. Endocannabinoids are also involved in the neuroendocrine control of reproduction functions through the modulation of stress, food intake, appetite, and sexual behaviour. Recently, new roles in sperm “startup” and gamete quality emerged and impairment of the physiological endocannabinoid tone and signalling has been reported in clinical cases of human infertility. Our hope is that this special issue may be important and timely since a deep knowledge of endocannabinoid system in reproduction might open new perspectives in clinical applications, pointing to endocannabinoid signalling as a novel target for correcting infertility, and for improving reproductive health in humans.

The papers submitted to this special issue in the International Journal of Endocrinology take into account the multifaceted aspects of reproduction. Basic and evolutionarily conserved mechanisms of endocannabinoid activity in reproduction have also been included. E. Cottone et al. submitted an extensive review on the role of the endocannabinoids in the central regulation of reproduction in nonmammalian vertebrates, especially fish and amphibian; they correlate the morphofunctional distribution of cannabinoid receptors to key molecules involved in the control of reproductive functions, such as Gonadotropin Releasing Hormone (GnRH), dopamine, aromatase, and pituitary gonadotropins.

The role of the endocannabinoid system as an ancient signalling system, that has been evolved over 500 million years, is highlighted in the paper of R. Chianese et al. that reports the presence of endocannabinoids in a nonmammalian model, the anuran amphibian *Rana esculenta*, and the functional crosstalk between these bioactive lipids and the GnRH system, shedding light on their different regulation in the brain and in the testis. In particular, a new role for vanilloid receptor emerged in the modulation of testicular GnRH system (both ligands and receptors) providing evidence that an opposite regulation occurs *via *type-1 cannabinoid receptor and vanilloid receptor signalling.

The paper of G. Cacciola et al. is a very interesting and comprehensive review on the pivotal role played by type-1 cannabinoid receptor in spermiogenesis and on its involvement in the chromatin remodelling process that might affect negatively the sperm quality. The emerging evidences on estrogen activity in sperm quality are deeply detailed in a knock-out animal model, opening new intriguing perspectives in the clinical practice for the treatment of male infertility.

The deep involvement of endocannabinoid signalling in driving the neurophysiological outcomes of mating behaviours has been reported in the research article submitted by J. M. Stuart et al. By means of lipidomic techniques, this group demonstrates that the levels of endocannabinoids, prostaglandins, and *N*-acylethanolamines rapidly change in specific brain areas in relationship to different mating strategies providing evidence that the endogenous cannabinoid systems is rapidly modified in response to changes in environment.

In the context of the signalling events of pregnancy, B. M. Fonseca et al. presented the most recent progress on the endocannabinoid regulatory functions during decidualization and placentation. They summarized that while the endocannabinoid machinery was found to be expressed in decidual and placental tissues, aberrant endocannabinoid signalling was associated with pregnancy disorders, highlighting the content that the endocannabinoid signalling is a potential player coordinating successful decidualization and placentation.

All of these papers have illustrated the potential regulatory interactions of sex steroid hormones with the endogenous cannabinoid system and how they allow reproduction to optimally function. The outstanding review by T. Ayakannu et al. that details the latest understandings of how sex steroids and the endogenous cannabinoid system work synergistically in a variety of cancers truly illustrates how the malfunctions of these signalling mechanisms can have dire effects. Using data from prostate, breast, and endometrial cancers, the review from Konje's group provides compelling evidence that to understand how to fix pathophysiology we must work towards understanding basic functioning physiology in the first place.

*Rosaria Meccariello* *Rosaria Meccariello* *Natalia Battista* *Natalia Battista* *Heather B. Bradshaw* *Heather B. Bradshaw* *Haibin Wang* *Haibin Wang*

